# Supplementary material for: Genome-Wide Identification, Expression, and Interaction Analysis of the Auxin Response Factor and AUX/IAA Gene Families in Vaccinium bracteatum
Source: Int J Mol Sci. 2024 Aug 1;25(15):8385. doi: 10.3390/ijms25158385 (PMC11312502; doi:10.3390/ijms25158385)
Supplement: Supplementary file 1 [file ijms-25-08385-s001.zip › S1Characterization of the ARF and IAA gene family.pdf]

Table S1-1 Characterization of the *ARF* gene family in *Vaccinium bracteatum* .

ID	Name	Location	CDS length (bp)	Protein			Exon
				Size (aa)	MW	pI	
1	*VaARF1-1*	LG02:9511674..9519181-	2041	679	75429.91	5.81	14
2	*VaARF1-2*	LG09:36711162..36721853+	2485	827	91876.94	5.67	14
3	*VaARF1-3*	LG08:40161872..40170246-	2386	794	88522.13	5.9	13
4	*VaARF2*	LG12:30583604..30590341-	2488	828	92476.82	6.21	14
5	*VaARF3*	LG11:1634690..1658521-	3643	1213	133143.06	7.32	21
6	*VaARF4*	LG06:31724240..31733991+	1882	626	69473.55	7.59	12
7	*VaARF5-1*	LG08:801128..809168+	3259	1085	121075.98	6.34	14
8	*VaARF5-2*	LG09:3417582..3424843+	2692	896	99795.22	5.46	14
9	*VaARF6*	LG06:28514131..28522625+	2656	884	97666.53	6.01	14
10	*VaARF9*	LG11:1588916..1604384-	1117	371	40970.78	8.74	11
11	*VaARF14-1*	LG11:28090727..28092378-	1102	366	40552.52	8.97	1
12	*VaARF14-2*	LG12:11767131..11768186+	1057	351	40189.42	6.54	1
13	*VaARF17*	LG02:27535040..27543430+	1723	573	62513.87	5.71	3
14	*VaARF18-1*	LG01:17518009..17523078+	2125	707	78263.29	6.23	14
15	*VaARF18-2*	LG06:31402261..31406753+	2053	683	75251.43	7.85	5
16	*VaARF19-1*	LG04:18689714..18693468-	2080	692	76254.04	6.38	5
17	*VaARF19-2*	LG09:31817340..31827285-	3247	1081	119563.01	6.18	15
18	*VaARF19-3*	LG09:31989605..31991121+	589	195	22120.92	5.45	3
19	*VaARF19-4*	LG09:31964263..31969027-	1390	462	52024.40	6.03	10
20	*VaARF19-5*	LG03:6942781..6947125-	1081	359	38716.52	8.11	6
21	*VaARF22*	LG07:30403271..30412840+	1171	389	44720.80	6.59	6
22	*VaARF28*	LG05:18017708..18026461+	2134	710	78212.38	7.05	12
23	*VaARF31-1*	LG07:36740709..36744843+	1141	379	43912.89	5.78	2
24	*VaARF31-2*	LG07:36722373..36723230+	859	285	33591.72	5.78	2
25	*VaARF31-3*	LG10:10522206..10528025+	1075	357	39853.74	6.24	3
26	*VaARF32*	LG02:3430645..3434320-	1504	500	57162.17	9.08	4

Table S1-2 Characterization of the *IAA* gene family in *Vaccinium bracteatum* .

ID	Name	Location	CDS length (bp)	Protein			Exon
				Size (aa)	MW	pI	
1	*VaIAA1*	LG05:42186252..42190991-	676	224	25065.34	5.06	5
2	*VaIAA5*	LG07:39274569..39276162+	577	191	21199.94	5.18	3
3	*VaIAA9*	LG03:35836698..35843807-	1117	371	39872.15	8.39	6
4	*VaIAA11*	LG07:37828066..37834023-	922	306	32547.34	7.67	5
5	*VaIAA13*	LG04:13382758..13387756+	841	279	29790.53	8.92	5
6	*VaIAA14-1*	LG04:9284381..9287508+	766	254	27710.7	6.64	5
7	*VaIAA14-2*	LG05:4021060..4024557+	760	252	27968.11	8.73	5
8	*VaIAA17*	LG03:3813271..3822584-	1819	605	66759.09	8.85	7
9	*VaIAA18*	LG12:34685385..34689437+	1093	363	40294.3	8.99	6
10	*VaIAA 20*	LG10:37848009..37851046-	685	227	25314.21	4.78	4
11	*VaIAA21*	LG08:8020121..8031743-	1405	467	50789.57	5.82	11
12	*VaIAA27-1*	LG05:42789015..42792771+	892	296	32516.73	8.74	5
13	*VaIAA27-3*	LG10:36474907..36479384-	928	308	32947.39	8.09	5
14	*VaIAA27-4*	LG12:34273712..34279439+	937	311	33794.24	8.06	5
15	*VaIAA26*	LG05:43015812..43020823+	1015	337	37067.73	8.12	6
16	*VaIAA32*	LG11:25919618..25921664+	574	190	21855.61	5.02	4
17	*VaIAA33*	LG01:857187..858513+	484	160	17720.05	7.84	2
