# Supplementary material for: Genome-Wide Identification, Expression, and Interaction Analysis of the Auxin Response Factor and AUX/IAA Gene Families in Vaccinium bracteatum
Source: Int J Mol Sci. 2024 Aug 1;25(15):8385. doi: 10.3390/ijms25158385 (PMC11312502; doi:10.3390/ijms25158385)
Supplement: Supplementary file 1 [file ijms-25-08385-s001.zip › S2 corresponding KaKs values.pdf]

Table S2-1 Identities of VaARF gene pairs and their corresponding Ka/Ks values

ID	Gene 1	Gene 2	Identity	Ka/Ks
1	*VaARF31-2*	*VaARF31-1*	85.7%	0.71202647
2	*VaARF9*	*VaARF3*	28.7%	0.617743222
3	*VaARF17*	*VaARF19-1*	53.6%	NaN
4	*VaARF1-3*	*VaARF1-2*	80.4%	0.173297715
	VaARF1-3	*VaARF6*	66.3%	0.094969143
5	*VaARF19-2*	*VaARF19-4*	68.0%	0.481872694
	VaARF19-2	*VaARF19-3*	19.0%	0.755533878

Table S2-2 Identities of VaIAA gene pairs and their corresponding Ka/Ks values

ID	Gene 1	Gene 2	Identity	Ka/Ks
1	*VaIAA9*	*VaIAA13*	52.4%	NaN
2	*VaIAA14-1*	*VaIAA14-2*	78.7%	0.109603753
3	*VaIAA11*	*VaIAA13*	63.5%	0.25553511
	VaIAA11	*VaIAA27-4*	59.4%	0.324252658
	VaIAA11	*VaIAA27-3*	56.7%	NaN
	VaIAA11	*VaIAA21*	47.7%	NaN
	VaIAA11	*VaIAA27-1*	54.4%	0.258428071
	VaIAA21	*VaIAA27-3*	60.3%	0.23009384
	VaIAA21	*VaIAA27-4*	47.6%	0.164086459
	VaIAA27-1	*VaIAA27-4*	74.6%	0.19208376
	VaIAA27-1	*VaIAA21*	49.6%	0.139085233
	VaIAA27-1	*VaIAA27-3*	70.2%	0.130015981
	VaIAA27-3	*VaIAA27-4*	66.8%	0.14893567
