# Supplementary material for: Genome-Wide Identification, Expression, and Interaction Analysis of the Auxin Response Factor and AUX/IAA Gene Families in Vaccinium bracteatum
Source: Int J Mol Sci. 2024 Aug 1;25(15):8385. doi: 10.3390/ijms25158385 (PMC11312502; doi:10.3390/ijms25158385)
Supplement: Supplementary file 1 [file ijms-25-08385-s001.zip › S3 Species id source.pdf]

TableS4-1 Source of ARF gene id in species

GeneID	Name	chromosome	organism
842268	*>NC_003070.9:21979289-21984433 ARF1*	chromosome=1	*Arabidopsis thaliana*
836321	*>NC_003076.8:24910191-24915395 ARF2*	chromosome=5	*Arabidopsis thaliana*
817014	*>NC_003071.7:10562688-10565296 ARF3*	chromosome=2	*Arabidopsis thaliana*
836166	*>NC_003076.8:c24312784-24308255 ARF4*	chromosome=5	*Arabidopsis thaliana*
839913	*>NC_003070.9:c10690988-10685822 ARF6*	chromosome=1	*Arabidopsis thaliana*
833672	*>NC_003076.8:14629453-14634454 ARF8*	chromosome=5	*Arabidopsis thaliana*
828498	*>NC_003075.7:12451143-12455369 ARF9*	chromosome=4	*Arabidopsis thaliana*
817382	*>NC_003071.7:12113889-12116957 ARF10*	chromosome=2	*Arabidopsis thaliana*
819264	*>NC_003071.7:19104632-19108486 ARF11*	chromosome=2	*Arabidopsis thaliana*
840331	*>NC_003070.9:c12511520-12508548 ARF12*	chromosome=1	*Arabidopsis thaliana*
840316	*>NC_003070.9:c12446764-12443547 ARF13*	chromosome=1	*Arabidopsis thaliana*
840450	*>NC_003070.9:13108634-13111700 ARF14*	chromosome=1	*Arabidopsis thaliana*
840447	*>NC_003070.9:c13085830-13082819 ARF15*	chromosome=1	*Arabidopsis thaliana*
829131	*>NC_003075.7:c14706354-14703065 ARF16*	chromosome=4	*Arabidopsis thaliana*
844120	*>NC_003070.9:29272074-29275507 ARF17*	chromosome=1	*Arabidopsis thaliana*
825356	*>NC_003074.8:22887889-22891455 ARF18*	chromosome=3	*Arabidopsis thaliana*
838505	*>NC_003070.9:c6633133-6627683 ARF19*	chromosome=1	*Arabidopsis thaliana*
840413	*>NC_003070.9:c12930523-12927303 ARF20*	chromosome=1	*Arabidopsis thaliana*
840344	*>NC_003070.9:12577722-12580824 ARF21*	chromosome=1	*Arabidopsis thaliana*
840341	*>NC_003070.9:12556005-12559082*	chromosome=1	*Arabidopsis thaliana*

840994	*ARF22*<br>*>NC_003070.9:c16673952-16672582*	chromosome=1	*Arabidopsis thaliana*
106376742	*ARF23*<br>*>NC_063444.1:31497606-31502540*	chromosome=C1	*Brassica napus*
111209353	*ARF1*<br>*>NC_063452.1:4775892-4780505*	chromosome=C9	*Brassica napus*
106431700	*ARF2-1*<br>*>NW_026016047.1:c97088-92673*	chromosome=Un	*Brassica napus*
106451191	*ARF2-2*<br>*>NC_063447.1:11243623-11246696*	chromosome=C4	*Brassica napus*
106371606	*ARF3*<br>*>NC_063452.1:c51748994-51744913*	chromosome=C9	*Brassica napus*
106371606	*ARF4-1*<br>*>NC_063452.1:c51748994-51744913*	chromosome=C9	*Brassica napus*
106411172	*ARF4-2*<br>*>NC_063450.1:c28032610-28028296*	chromosome=C7	*Brassica napus*
106433157	*ARF5-1*<br>*>NC_063451.1:29762244-29766388*	chromosome=C8	*Brassica napus*
106410709	*ARF5-2*<br>*>NC_063448.1:c21931507-21926639*	chromosome=C5	*Brassica napus*
106418427	*ARF6-1*<br>*>NC_063446.1:c58436980-58431789*	chromosome=C3	*Brassica napus*
106425782	*ARF6-2*<br>*>NC_063445.1:c4365955-4360474*	chromosome=C2	*Brassica napus*
106376592	*ARF7-1*<br>*>NC_063452.1:53715686-53721289*	chromosome=C9	*Brassica napus*
106376592	*ARF7-2*<br>*>NC_063452.1:53715686-53721289*	chromosome=C9	*Brassica napus*
106435561	*ARF7-3*<br>*>NC_063447.1:c41961727-41957061*	chromosome=C4	*Brassica napus*
106396551	*ARF8*<br>*>NC_063447.1:c18099405-18096477*	chromosome=C4	*Brassica napus*
106414365	*ARF10*<br>*>NC_063447.1:c1334593-1331240*	chromosome=C4	*Brassica napus*
106411312	*ARF11*<br>*>NC_063450.1:c51727904-51724702*	chromosome=C7	*Brassica napus*
106354590	*ARF16*<br>*>NC_063449.1:46855817-46858985*	chromosome=C6	*Brassica napus*
106354590	*ARF17-1*<br>*>NC_063449.1:46855817-46858985*	chromosome=C6	*Brassica napus*
106396799	*ARF17-2*<br>*>NC_063447.1:c29536273-29533115*	chromosome=C4	*Brassica napus*
106431859	*ARF18*<br>*>NC_063451.1:c29427101-29422357*	chromosome=C8	*Brassica napus*

	ARF19-1		
106401590	*>NC_063448.1:c10603452-10598637 ARF19-2*	chromosome=C5	*Brassica napus*
106401590	*>NC_063448.1:c10603452-10598637 ARF19-3*	chromosome=C5	*Brassica napus*
106414636	*>NC_063452.1:c1694980-1691939 ARF21-1*	chromosome=C9	*Brassica napus*
106395600	*>NC_063447.1:c10580147-10575398 ARF21-2*	chromosome=C4	*Brassica napus*
106395600	*>NC_063447.1:c10580147-10575398 ARF21-3*	chromosome=C4	*Brassica napus*
106363036	*>NC_063452.1:27281849-27290091 ARF21-4*	chromosome=C9	*Brassica napus*
100819023	*>NC_038252.2:c31960-25203 ARF1*	chromosome=16	*Glycine max*
100802387	*>NC_038255.2:c46706392-46694976 ARF2*	chromosome=19	*Glycine max*
100802387	*>NC_038255.2:c46706392-46694976 ARF2-2*	chromosome=19	*Glycine max*
100797082	*>NC_016091.4:c46143156-46138356 ARF2B*	chromosome=4	*Glycine max*
100803498	*>NC_038242.2:13626389-13631496 ARF2B-2*	chromosome=6	*Glycine max*
100815956	*>NC_038241.2:38470691-38475799 ARF2B-3*	chromosome=5	*Glycine max*
100796970	*>NC_038243.2:c37452886-37447266 ARF3*	chromosome=7	*Glycine max*
100808119	*>NC_038249.2:28226412-28232396 ARF3-2*	chromosome=13	*Glycine max*
100812909	*>NC_038248.2:c34114756-34108270 ARF4*	chromosome=12	*Glycine max*
100816621	*>NC_038247.2:c11587768-11580926 ARF4-2*	chromosome=11	*Glycine max*
100813003	*>NC_038253.2:c41140740-41134985 ARF5*	chromosome=17	*Glycine max*
100796447	*>NC_038251.2:c6977283-6970484 ARF6*	chromosome=15	*Glycine max*
100798719	*>NC_038249.2:32864285-32871165 ARF6-2*	chromosome=13	*Glycine max*
100806203	*>NC_038244.2:c7683572-7676757 ARF6-3*	chromosome=8	*Glycine max*
100809105	*>NC_016089.4:48226289-48235338 ARF6-4*	chromosome=2	*Glycine max*
100814479	*>NC_038254.2:4068571-4076048 ARF8*	chromosome=18	*Glycine max*

100795763	*>NC_038252.2:c2248090-2242910*<br>*ARF9*	chromosome=16	*Glycine max*
100799088	*>NC_038243.2:15844228-15849774*<br>*ARF9-2*	chromosome=7	*Glycine max*
100803911	*>NC_016088.4:c35192417-35187514*<br>*ARF9-3*	chromosome=1	*Glycine max*
100803911	*>NC_016088.4:c35192417-35187514*<br>*ARF9-4*	chromosome=1	*Glycine max*
100804933	*>NC_038250.2:42041660-42045868*<br>*ARF17*	chromosome=14	*Glycine max*
100805638	*>NC_038249.2:18613985-18618480*<br>*ARF17-2*	chromosome=13	*Glycine max*
102662128	*>NC_016091.4:50908654-50912930*<br>*ARF17-3*	chromosome=4	*Glycine max*
100796522	*>NC_038246.2:c4809586-4804492*<br>*ARF18*	chromosome=10	*Glycine max*
100803053	*>NC_038246.2:44358919-44362461*<br>*ARF18-2*	chromosome=10	*Glycine max*
100803053	*>NC_038246.2:44358919-44362461*<br>*ARF18-3*	chromosome=10	*Glycine max*
100808472	*>NC_038249.2:41405819-41408904*<br>*ARF18-4*	chromosome=13	*Glycine max*
100814686	*>NC_038248.2:c5840503-5836804*<br>*ARF18-5*	chromosome=12	*Glycine max*
100818234	*>NC_038256.2:c41747651-41744507*<br>*ARF18-6*	chromosome=20	*Glycine max*
100820503	*>NC_016090.4:46452032-46456633*<br>*ARF18-7*	chromosome=3	*Glycine max*
100803302	*>NC_038241.2:c40129887-40123104*<br>*ARF19*	chromosome=5	*Glycine max*
100804628	*>NC_038245.2:c7444983-7436568*<br>*ARF19-2*	chromosome=9	*Glycine max*
100805456	*>NC_038249.2:c21620984-21613615*<br>*ARF19-3*	chromosome=13	*Glycine max*
100815277	*>NC_038251.2:c17530155-17521996*<br>*ARF19-4*	chromosome=15	*Glycine max*
100817780	*>NC_016088.4:c300406-294451*<br>*ARF19-5*	chromosome=1	*Glycine max*
100816054	*>NC_038253.2:3563790-3571666*<br>*ARF19-6*	chromosome=17	*Glycine max*
102665969	*>NC_038243.2:15851185-15855395*<br>*ARF21*	chromosome=7	*Glycine max*
107896654	*>NC_053433.1:93377963-93384770*<br>*ARF1*	chromosome=A10	*Gossypium hirsutum*

107944673	*>NC_053447.1:c3432282-3426731*<br>*ARF2*	chromosome=D11	*Gossypium hirsutum*
107925223	*>NC_053433.1:c2661861-2657445*<br>*ARF3-1*	chromosome=A10	*Gossypium hirsutum*
107941725	*>NC_053428.1:15636662-15642542*<br>*ARF3-2*	chromosome=A05	*Gossypium hirsutum*
107889252	*>NC_053432.1:c67659309-67653038*<br>*ARF4-1*	chromosome=A09	*Gossypium hirsutum*
107891491	*>NC_053445.1:c39309567-39304434*<br>*ARF4-2*	chromosome=D09	*Gossypium hirsutum*
107906725	*>NC_053441.1:6280651-6286312*<br>*ARF4-3*	chromosome=D05	*Gossypium hirsutum*
107918011	*>NC_053424.1:22786326-22792278*<br>*ARF5-1*	chromosome=A01	*Gossypium hirsutum*
107922066	*>NC_053437.1:17965392-17971445*<br>*ARF5-2*	chromosome=D01	*Gossypium hirsutum*
107888470	*>NC_053432.1:c1926225-1918551*<br>*ARF6-1*	chromosome=A09	*Gossypium hirsutum*
107905980	*>NC_053441.1:12928923-12935947*<br>*ARF6-2*	chromosome=D05	*Gossypium hirsutum*
107916462	*>NC_053446.1:c4158095-4150649*<br>*ARF6-3*	chromosome=D10	*Gossypium hirsutum*
107937515	*>NC_053433.1:c4437828-4430562*<br>*ARF6-4*	chromosome=A10	*Gossypium hirsutum*
107939032	*>NC_053445.1:c1925440-1917768*<br>*ARF6-5*	chromosome=D09	*Gossypium hirsutum*
107945439	*>NC_053448.1:15616584-15626800*<br>*ARF6-6*	chromosome=D12	*Gossypium hirsutum*
107958298	*>NC_053428.1:14214000-14221067*<br>*ARF6-7*	chromosome=A05	*Gossypium hirsutum*
107901036	*>NC_053442.1:c15273387-15265688*<br>*ARF7-1*	chromosome=D06	*Gossypium hirsutum*
107962189	*>NC_053429.1:c22327063-22319271*<br>*ARF7-2*	chromosome=A06	*Gossypium hirsutum*
107935696	*>NC_053448.1:8525075-8541244*<br>*ARF8-1*	chromosome=D12	*Gossypium hirsutum*
107954826	*>NC_053443.1:46635969-46648574*<br>*ARF8-2*	chromosome=D07	*Gossypium hirsutum*
107904460	*>NC_053447.1:2016777-2021489*<br>*ARF9-1*	chromosome=D11	*Gossypium hirsutum*
107951171	*>NC_053425.1:c45241273-45236531*<br>*ARF9-2*	chromosome=A02	*Gossypium hirsutum*
107959020	*>NC_053428.1:c9724258-9719889*<br>*ARF10*	chromosome=A05	*Gossypium hirsutum*

107952962	*>NC_053430.1:c30212480-30204964<br>ARF11*	chromosome=A07	*Gossypium hirsutum*
107891134	*>NC_053445.1:45155154-45159483<br>ARF18-1*	chromosome=D09	*Gossypium hirsutum*
107904267	*>NC_053428.1:110556005-110559178<br>ARF18-2*	chromosome=A05	*Gossypium hirsutum*
107904267	*>NC_053428.1:110556005-110559178<br>ARF18-3*	chromosome=A05	*Gossypium hirsutum*
107924220	*>NC_053447.1:9150980-9155641<br>ARF18-4*	chromosome=D11	*Gossypium hirsutum*
100814686	*>NC_053434.1:9817641-9823950<br>ARF18-5*	chromosome=A11	*Gossypium hirsutum*
107930156	*>NC_053436.1:c111227700-111223319<br>ARF18-6*	chromosome=A13	*Gossypium hirsutum*
107936074	*>NC_053449.1:c64704695-64701463<br>ARF18-7*	chromosome=D13	*Gossypium hirsutum*
107944087	*>NC_053433.1:111452668-111455829<br>ARF18-8*	chromosome=A10	*Gossypium hirsutum*
107954481	*>NC_053443.1:c22849200-22844671<br>ARF18-9*	chromosome=D07	*Gossypium hirsutum*
107904307	*>NC_053428.1:c110202086-110194730<br>ARF19-1*	chromosome=A05	*Gossypium hirsutum*
107907087	*>NC_053441.1:c3041710-3034564<br>ARF19-2*	chromosome=D05	*Gossypium hirsutum*
107929914	*>NC_053430.1:c1835829-1829380<br>ARF19-3*	chromosome=A07	*Gossypium hirsutum*
107958833	*>NC_053428.1:c3578272-3571243<br>ARF19-4*	chromosome=A05	*Gossypium hirsutum*

Table S4-2 Source of IAA gene id in species

GeneID	Name	chromosome	organism
827103	*NC_003075.7:8360996-8362033<br>AtIAA1*	chromosome=4	*Arabidopsis thaliana*
821877	*NC_003074.8:c8182095-8180646<br>AtIAA2*	chromosome=3	*Arabidopsis thaliana*
838128	*NC_003070.9:c5366497-5365512<br>AtIAA5*	chromosome=1	*Arabidopsis thaliana*
841717	*NC_003070.9:c19673652-19672476<br>AtIAA6*	chromosome=1	*Arabidopsis thaliana*
821879	*NC_003074.8:8194606-8197161*	chromosome=3	*Arabidopsis thaliana*

816798	*AtIAA7*<br>*NC_003071.7:9636346-9638761*	chromosome=2	*Arabidopsis thaliana*
836693	*AtIAA8*<br>*NC_003076.8:26253408-26256544*	chromosome=5	*Arabidopsis thaliana*
839290	*AtIAA9*<br>*NC_003070.9:1059470-1061209*	chromosome=1	*Arabidopsis thaliana*
828982	*AtIAA10*<br>*NC_003075.7:14142063-14143969*	chromosome=4	*Arabidopsis thaliana*
839495	*AtIAA11*<br>*NC_003070.9:1240294-1242157*	chromosome=1	*Arabidopsis thaliana*
817894	*AtIAA12*<br>*NC_003071.7:c14116089-14113025*	chromosome=2	*Arabidopsis thaliana*
827102	*AtIAA13*<br>*NC_003075.7:c8350263-8347822*	chromosome=4	*Arabidopsis thaliana*
844379	*AtIAA14*<br>*NC_003070.9:c30222769-30221629*	chromosome=1	*Arabidopsis thaliana*
819633	*AtIAA15*<br>*NC_003074.8:c1290891-1288155*	chromosome=3	*Arabidopsis thaliana*
841623	*AtIAA16*<br>*NC_003070.9:19305081-19307518*	chromosome=1	*Arabidopsis thaliana*
820793	*AtIAA18*<br>*NC_003074.8:5264001-5265695*	chromosome=3	*Arabidopsis thaliana*
819313	*AtIAA19*<br>*NC_003071.7:19307714-19309071*	chromosome=2	*Arabidopsis thaliana*
832658	*AtIAA20*<br>*NC_003076.8:9033204-9035140*	chromosome=5	*Arabidopsis thaliana*
829361	*AtIAA28*<br>*NC_003075.7:15583332-15584903*	chromosome=4	*Arabidopsis thaliana*
825383	*AtIAA29*<br>*NC_003074.8:22995686-22996809*	chromosome=3	*Arabidopsis thaliana*
821026	*AtIAA30*<br>*NC_003074.8:c6021147-6019973*	chromosome=3	*Arabidopsis thaliana*
814648	*AtIAA31*<br>*NC_003071.7:117969-120095*	chromosome=2	*Arabidopsis thaliana*
835848	*AtIAA32*<br>*NC_003076.8:23269914-23271092*	chromosome=5	*Arabidopsis thaliana*
838070	*AtIAA33*<br>*NC_003070.9:c5183413-5181991*	chromosome=1	*Arabidopsis thaliana*
100816483	*AtIAA34*<br>*NC_038241.2:c33049317-33040449*	chromosome=5	*Glycine max*
100818083	*GmIAA1*<br>*NC_038249.2:22064365-22065943*	chromosome=13	*Glycine max*
100785203	*GmIAA4*<br>*NC_038245.2:43629808-43634225*	chromosome=9	*Glycine max*

	GmIAA8		
100789251	*NC_038244.2:c35401397-35396004*<br>*GmIAA9*	chromosome=8	*Glycine max*
100815447	*NC_038250.2:45804839-45808997*<br>*GmIAA9-2*	chromosome=14	*Glycine max*
100818881	*NC_016089.4:42374250-42379102*<br>*GmIAA9-3*	chromosome=2	*Glycine max*
100789143	*NC_016090.4:39347977-39352756*<br>*GmIAA10*	chromosome=3	*Glycine max*
100527033	*NC_038246.2:3544019-3547982*<br>*GmIAA11*	chromosome=10	*Glycine max*
100779400	*NC_038249.2:23101490-23105532*<br>*GmIAA11-2*	chromosome=13	*Glycine max*
100810762	*NC_038256.2:c44583674-44581204*<br>*GmIAA14*	chromosome=20	*Glycine max*
100793822	*NC_038243.2:1197319-1200815*<br>*GmIAA16*	chromosome=7	*Glycine max*
100796888	*NC_016090.4:45594796-45597342*<br>*GmIAA16-2*	chromosome=3	*Glycine max*
100803986	*NC_038255.2:49755887-49758397*<br>*GmIAA16-3*	chromosome=19	*Glycine max*
100815098	*NC_038251.2:c1004189-1002446*<br>*GmIAA17*	chromosome=15	*Glycine max*
100783140	*NC_038255.2:c47909746-47908463*<br>*GmIAA20*	chromosome=19	*Glycine max*
100818376	*NC_016090.4:c43888126-43886799*<br>*GmIAA20-2*	chromosome=3	*Glycine max*
547784	*NC_038244.2:16886498-16890948*<br>*GmIAA22*	chromosome=8	*Glycine max*
100791342	*NC_038243.2:c2697824-2694045*<br>*GmIAA22-2*	chromosome=7	*Glycine max*
100792134	*NC_038255.2:c42617131-42615456*<br>*GmIAA22E*	chromosome=19	*Glycine max*
100809479	*NC_016090.4:c38565259-38563579*<br>*GmIAA22E-2*	chromosome=3	*Glycine max*
100799244	*NC_038249.2:c43626945-43622952*<br>*GmIAA26*	chromosome=13	*Glycine max*
100804593	*NC_038251.2:1574242-1577516*<br>*GmIAA26-2*	chromosome=15	*Glycine max*
100785733	*NC_016088.4:c4250293-4248466*<br>*GmIAA27*	chromosome=1	*Glycine max*
100795203	*NC_038243.2:c1446991-1443707*<br>*GmIAA27-2*	chromosome=7	*Glycine max*
100803754	*NC_038245.2:42650566-42654112*	chromosome=9	*Glycine max*

	27-3		
100806699	*NC_038249.2:c43840367-43837735 GmIAA27-4*	chromosome=13	*Glycine max*
547965	*NC_038255.2:42630467-42634596 GmIAA28*	chromosome=19	*Glycine max*
100783359	*NC_038242.2:5148460-5150116 GmIAA28-2*	chromosome=6	*Glycine max*
100810013	*NC_016090.4:38578277-38581824 GmIAA28-3*	chromosome=3	*Glycine max*
100808466	*NC_038249.2:c26852681-26851266 GmIAA29*	chromosome=13	*Glycine max*
100781660	*NC_038246.2:c37370719-37369072 GmIAA30*	chromosome=10	*Glycine max*
100784866	*NC_016089.4:761009-763376 GmIAA30-2*	chromosome=2	*Glycine max*
100777024	*NC_038246.2:49349090-49350631 GmIAA32*	chromosome=10	*Glycine max*
100793397	*NC_038256.2:c36328381-36326869 GmIAA32-2*	chromosome=20	*Glycine max*
547949	*NC_038246.2:41470294-41472900 GmIAA50*	chromosome=10	*Glycine max*
106353873	*NC_063449.1:c37728432-37725724 BnIAA1*	chromosome=C6	*Brassica napus*
106353997	*NC_063440.1:23149361-23152151 BnIAA1-2*	chromosome=A7	*Brassica napus*
106367504	*NC_063448.1:37930830-37931988 BnIAA2*	chromosome=C5	*Brassica napus*
106416441	*NC_063446.1:c32019148-32017790 BnIAA2-2*	chromosome=C3	*Brassica napus*
106366639	*NC_063452.1:20228529-20229660 BnIAA4*	chromosome=C9	*Brassica napus*
106405046	*NC_063449.1:c9546587-9544243 BnIAA6*	chromosome=C6	*Brassica napus*
106367506	*NC_063448.1:c37887109-37884790 BnIAA7*	chromosome=C5	*Brassica napus*
106439612	*NC_063436.1:19840773-19842979 BnIAA7-2*	chromosome=A3	*Brassica napus*
106451788	*NC_063438.1:c18852727-18850468 BnIAA7-3*	chromosome=A5	*Brassica napus*
106348109	*NC_063446.1:c37889088-37886607 BnIAA9*	chromosome=C3	*Brassica napus*
106415790	*NC_063452.1:6295041-6297087 BnIAA9-2*	chromosome=C9	*Brassica napus*
106402229	*NC_063448.1:1247677-1250079*	chromosome=C5	*Brassica napus*

	BnIAA10		
106399391	*NC_063448.1:1475352-1477439*<br>*BnIAA12*	chromosome=C5	*Brassica napus*
106388029	*NC_063446.1:c10538997-10537236*<br>*BnIAA13*	chromosome=C3	*Brassica napus*
106451148	*NC_063447.1:11698933-11700543*<br>*BnIAA13-2*	chromosome=C4	*Brassica napus*
106427417	*NC_063448.1:54993728-54995741*<br>*BnIAA16*	chromosome=C5	*Brassica napus*
106449503	*NC_063449.1:48086742-48088186*<br>*BnIAA15*	chromosome=C6	*Brassica napus*
106369247	*NC_063442.1:c44280301-44277972*<br>*BnIAA17*	chromosome=A9	*Brassica napus*
106403322	*NC_063449.1:8511787-8513874*<br>*BnIAA18*	chromosome=C6	*Brassica napus*
106444036	*NC_063436.1:17928157-17929700*<br>*BnIAA19*	chromosome=A3	*Brassica napus*
106454444	*NC_063437.1:21895876-21899954*<br>*BnIAA20*	chromosome=A4	*Brassica napus*
106358145	*NC_063446.1:c28398238-28396361*<br>*BnIAA26*	chromosome=C3	*Brassica napus*
106439439	*NC_063436.1:c18219347-18217411*<br>*BnIAA26-2*	chromosome=A3	*Brassica napus*
106452119	*NC_063448.1:47452275-47454271*<br>*BnIAA26-3*	chromosome=C5	*Brassica napus*
106452205	*NW_026016601.1:35956-37973*<br>*BnIAA26-4*	chromosome=Un	*Brassica napus*
106352191	*NC_063450.1:c43171928-43170365*<br>*BnIAA28*	chromosome=C7	*Brassica napus*
106444457	*NC_063452.1:c3643091-3641551*<br>*BnIAAA28-2*	chromosome=C9	*Brassica napus*
106356870	*NC_063440.1:18666147-18667409*<br>*BnIAA30*	chromosome=A7	*Brassica napus*
111211370	*NC_063449.1:29933145-29934650*<br>*BnIAA30-2*	chromosome=C6	*Brassica napus*
106386635	*NC_063446.1:c28837754-28836223*<br>*BnIAA31*	chromosome=C3	*Brassica napus*
106444285	*NC_063448.1:c7597967-7596265*<br>*BnIAA34*	chromosome=C5	*Brassica napus*
107918102	*NC_053435.1:c92743879-92731307*<br>*GhIAA1*	chromosome=A12	*Gossypium hirsutum*
107946309	*NC_053448.1:c49194472-49182133*<br>*GhIAA1-2*	chromosome=D12	*Gossypium hirsutum*
107909235	*NC_053431.1:c126327693-126324309*	chromosome=A08	*Gossypium hirsutum*

	GhIAA1-3		
107933441	*NC_053446.1:37804080-37805635* *GhIAA4*	chromosome=D10	*Gossypium hirsutum*
107940165	*NC_053425.1:c4304941-4303366* *GhIAA4-2*	chromosome=A02	*Gossypium hirsutum*
107905200	*NC_053441.1:c19698994-19694769* *GhIAA8*	chromosome=D05	*Gossypium hirsutum*
107914352	*NC_053446.1:c13733379-13730042* *GhIAA9*	chromosome=D10	*Gossypium hirsutum*
107941326	*NC_053442.1:c3363312-3359412* *GhIAA9-2*	chromosome=D06	*Gossypium hirsutum*
107950498	*NC_053439.1:c3799623-3795670* *GhIAA9-3*	chromosome=D03	*Gossypium hirsutum*
107923990	*NC_053447.1:13847240-13850789* *GhIAA11*	chromosome=D11	*Gossypium hirsutum*
107952058	*NC_053433.1:c25246477-25244309* *GhIAA13*	chromosome=A10	*Gossypium hirsutum*
107940169	*NC_053425.1:4322278-4324224* *GhIAA14*	chromosome=A02	*Gossypium hirsutum*
107889565	*NC_053434.1:c107026545-107024376* *GhIAA16*	chromosome=A11	*Gossypium hirsutum*
107906498	*NC_053441.1:c10971726-10970333* *GhIAA16-2*	chromosome=D05	*Gossypium hirsutum*
107926842	*NC_053447.1:c57512777-57510673* *GhIAA16-3*	chromosome=D11	*Gossypium hirsutum*
107906133	*NC_053441.1:11507427-11510901* *GhIAA18*	chromosome=D05	*Gossypium hirsutum*
107901319	*NC_053442.1:8441896-8443121* *GhIAA20*	chromosome=D06	*Gossypium hirsutum*
107906499	*NC_053441.1:c10981652-10979993* *GhIAA22*	chromosome=D05	*Gossypium hirsutum*
107920280	*NC_053449.1:57508313-57509525* *GhIAA22B*	chromosome=D13	*Gossypium hirsutum*
107889557	*NC_053434.1:107056127-107057447* *GhIAA22D-1*	chromosome=A11	*Gossypium hirsutum*
107915183	*NC_053444.1:9058321-9059737* *GhIAA22D-2*	chromosome=D08	*Gossypium hirsutum*
107929024	*NC_053445.1:c52312884-52311435* *GhIAA22D-3*	chromosome=D09	*Gossypium hirsutum*
107928438	*NC_053432.1:c82983642-82982152* *GhIAA22D-4*	chromosome=A09	*Gossypium hirsutum*
107941656	*NC_053443.1:c55676875-55675361* *GhIAA22D-5*	chromosome=D07	*Gossypium hirsutum*
107955471	*NC_053430.1:c95112158-95110752*	chromosome=A07	*Gossypium hirsutum*

	GhIAA22D-6		
107897354	*NC_053433.1:c7893829-7890658* *GhIAA26*	chromosome=A10	*Gossypium hirsutum*
107913884	*NC_053446.1:c6923373-6920262* *GhIAA26-2*	chromosome=D10	*Gossypium hirsutum*
107937353	*NC_053429.1:126384190-126387227* *GhIAA26-3*	chromosome=A06	*Gossypium hirsutum*
107907196	*NC_053441.1:2309412-2311906* *GhIAA27*	chromosome=D05	*Gossypium hirsutum*
107950694	*NC_053439.1:c12779662-12775979* *GhIAA27-2*	chromosome=D03	*Gossypium hirsutum*
107922883	*NC_053447.1:12932685-12935413* *GhIAA28*	chromosome=D11	*Gossypium hirsutum*
107924370	*NC_053434.1:14866196-14869342* *GhIAA28-2*	chromosome=A11	*Gossypium hirsutum*
107955469	*NC_053430.1:95125547-95128105* *GhIAA28-3*	chromosome=A07	*Gossypium hirsutum*
