# Supplementary material for: Genome-Wide Identification, Expression, and Interaction Analysis of the Auxin Response Factor and AUX/IAA Gene Families in Vaccinium bracteatum
Source: Int J Mol Sci. 2024 Aug 1;25(15):8385. doi: 10.3390/ijms25158385 (PMC11312502; doi:10.3390/ijms25158385)
Supplement: Supplementary file 1 [file ijms-25-08385-s001.zip › S4 Cis-acting element schedule.pdf]

Table3.1　cis-acting elements of VaARF gene family

Gene	CREs
VaARF1-2	MeJA-responsiveness
	light responsive elements
	elements of growth and develpoment
	MYB　binding site involved in drought-inducibility
	hormone responsive elements
VaARF18-1	light responsive elements
	low-temperature responsiveness
	MeJA-responsiveness
VaARF19-2	elements of growth and develpoment
	light responsive elements
	MYB　binding site involved in drought-inducibility
	low-temperature responsiveness
	hormone responsive elements
VaARF19-5	light responsive elements
	stress responsive elements
	MeJA-responsiveness
	low-temperature responsiveness
	auxin responsiveness
	hormone responsive elements
	elements of growth and develpoment
VaARF19-4	light responsive elements
	MeJA-responsiveness
	hormone responsive elements
	low-temperature responsiveness
	MYB　binding site involved in drought-inducibility
VaARF14-1	hormone responsive elements
	MYB　binding site involved in drought-inducibility
	light responsive elements
VaARF18-2	MeJA-responsiveness
	elements of growth and develpoment
	auxin responsiveness
	MYB　binding site involved in drought-inducibility
	hormone responsive elements

	light responsive elements
	gibberellin-responsive element
VaARF17	light responsive elements
	hormone responsive elements
	elements of growth and develpoment
	MeJA-responsiveness
	auxin responsiveness
VaARF1-1	light responsive elements
	elements of growth and develpoment
	hormone responsive elements
	MeJA-responsiveness
	MYB binding site involved in drought-inducibility
	auxin responsiveness
VaARF31-2	light responsive elements
	MeJA-responsiveness
	elements of growth and develpoment
	MYB binding site involved in drought-inducibility
	auxin responsiveness
	hormone responsive elements
VaARF9	light responsive elements
	elements of growth and develpoment
	MeJA-responsiveness
	auxin responsiveness
	hormone responsive elements
	MYB binding site involved in drought-inducibility
VaARF22	low-temperature responsiveness
	light responsive elements
	elements of growth and develpoment
	auxin responsiveness
	hormone responsive elements
	MYB binding site involved in drought-inducibility
VaARF2	light responsive elements
	MeJA-responsiveness
	auxin responsiveness
	MYB binding site involved in drought-inducibility
VaARF19-1	elements of growth and develpoment
	MeJA-responsiveness

	MYB binding site involved in drought-inducibility
	hormone responsive elements
	light responsive elements
	low-temperature responsiveness
	gibberellin-responsive element
	light responsive elements
VaARF5-2	light responsive elements
	elements of growth and develpoment
	hormone responsive elements
	MYB binding site involved in drought-inducibility
	stress responsive elements
	gibberellin-responsive element
VaARF31-1	elements of growth and develpoment
	light responsive elements
	low-temperature responsiveness
	hormone responsive elements
	MYB binding site involved in drought-inducibility
ARF6	low-temperature responsiveness
	MYB binding site involved in drought-inducibility
	light responsive elements
	hormone responsive elements
	elements of growth and develpoment
	MeJA-responsiveness
	gibberellin-responsive element
VaARF1-3	elements of growth and develpoment
	light responsive elements
	MeJA-responsiveness
	hormone responsive elements
	auxin responsiveness
VaARF28	MYB binding site involved in drought-inducibility
	hormone responsive elements
	light responsive elements
	MeJA-responsiveness
VaARF14-2	light responsive elements
	hormone responsive elements
	low-temperature responsiveness
VaARF31-3	auxin responsiveness

	light responsive elements
	MeJA-responsiveness
	hormone responsive elements
	low-temperature responsiveness
VaARF5-1	light responsive elements
	MeJA-responsiveness
	MYB binding site involved in drought-inducibility
	hormone responsive elements
VaARF19-3	light responsive elements
	MeJA-responsiveness
	MYB binding site involved in drought-inducibility
	hormone responsive elements
	low-temperature responsiveness
VaARF4	light responsive elements
	low-temperature responsiveness
	hormone responsive elements
	MYB binding site involved in drought-inducibility
	elements of growth and develpoment
	gibberellin-responsive element
VaARF32	auxin responsiveness
	elements of growth and develpoment
	light responsive elements
	MeJA-responsiveness
	low-temperature responsiveness
	hormone responsive elements
VaARF3	elements of growth and develpoment
	MeJA-responsiveness
	light responsive elements
	hormone responsive elements
	auxin responsiveness
	MYB binding site involved in drought-inducibility
	gibberellin-responsive element

Table3.2 cis-acting elements of VaIAA gene family

Gene	CREs
VaIAA27-1	MYB binding site involved in drought-inducibility
	hormone responsive elements
	light responsive elements
	stress responsive elements
	MYB binding site involved in light responsiveness
VaIAA13	light responsive elements
	stress responsive elements
	elements of growth and develpoment
	hormone responsive elements
	MeJA-responsiveness
VaIAA27-4	light responsive elements
	stress responsive elements
	hormone responsive elements
	MeJA-responsiveness
	auxin responsiveness
VaIAA20	hormone responsive elements
	stress responsive elements
	light responsive elements
	elements of growth and develpoment
VaIAA11	light responsive elements
	hormone responsive elements
	elements of growth and develpoment
	stress responsive elements
	MYB binding site involved in drought-inducibility
	MeJA-responsiveness
VaIAA14-2	light responsive elements
	stress responsive elements
	hormone responsive elements
	MeJA-responsiveness
VaIAA26	stress responsive elements

	light responsive elements
	auxin responsiveness
	elements of growth and develpoment
	hormone responsive elements
VaIAA21	stress responsive elements
	light responsive elements
	low-temperature responsiveness
	MeJA-responsiveness
	hormone responsive elements
	MYB binding site involved in drought-inducibility
VaIAA17	hormone responsive elements
	light responsive elements
	auxin responsiveness
	stress responsive elements
	elements of growth and develpoment
	MYB binding site involved in drought-inducibility
VaIAA33	MeJA-responsiveness
	light responsive elements
	hormone responsive elements
	stress responsive elements
	elements of growth and develpoment
VaIAA32	hormone responsive elements
	light responsive elements
	elements of growth and develpoment
	stress responsive elements
	auxin responsiveness
	MeJA-responsiveness
	MYB binding site involved in drought-inducibility
VaIAA18	MYB binding site involved in drought-inducibility
	stress responsive elements
	light responsive elements
	low-temperature responsiveness
	MeJA-responsiveness
	MYB binding site involved in light responsiveness
	elements of growth and develpoment
VaIAA9	hormone responsive elements
	stress responsive elements

	light responsive elements
	MeJA-responsiveness
	MYB binding site involved in drought-inducibility
VaIAA27-2	stress responsive elements
	light responsive elements
	elements of growth and develpoment
	hormone responsive elements
	auxin responsiveness
VaIAA1	light responsive elements
	stress responsive elements
	MYB binding site involved in drought-inducibility
VaIAA14-1	hormone responsive elements
	defense and stress responsiveness
	light responsive elements
VaIAA27-3	auxin responsiveness
	MeJA-responsiveness
	light responsive elements
	defense and stress responsiveness
VaIAA5	light responsive elements
	MeJA-responsiveness
